# Application of Anionic Hydrogels from Date Palm Waste for Dye Adsorption in Wastewater Treatment

**DOI:** 10.3390/gels10100617

**Published:** 2024-09-26

**Authors:** Farid Fadhillah, Abdulrahman G. Alhamzani, Khaled Bin Bandar, Abdullah Alshamari, Saad Aljlil, Abdelrahman G. Gadallah, M. A. Habib, Mortaga M. Abou-Krisha, Mona A. Abdel-Fatah

**Affiliations:** 1 Chemical Engineering Department, College of Engineering, Imam Mohammad Ibn Saud Islamic University (IMSIU), Riyadh 11432, Saudi Arabia; fffadhillah@imamu.edu.sa (F.F.); agadallah@imamu.edu.sa (A.G.G.); 2 Department of Chemistry, Imam Mohammad Ibn Saud Islamic University (IMSIU), Riyadh 11623, Saudi Arabia; agalhamzani@imamu.edu.sa (A.G.A.); mahabib@imamu.edu.sa (M.A.H.); mmaboukrisha@imamu.edu.sa (M.M.A.-K.); 3 Water Management & Treatment Institute, King Abdulaziz City for Science and Technology (KACST), Riyadh 11442, Saudi Arabia; kbandar@kacst.gov.sa (K.B.B.); akshamari@kacst.gov.sa (A.A.); saljlil@kacst.gov.sa (S.A.); 4 Chemical Engineering and Pilot Plant Department, National Research Centre, Engineering and Renewable Energy Research Institute, Giza 12622, Egypt; 5 Chemistry of Tanning Materials and Leather Technology Department, National Research Centre, Chemical Industries Institute, Giza 12622, Egypt

**Keywords:** dye wastewater treatment, cellulose nanofiber, bio-adsorbent, date palm leaves, methylene blue

## Abstract

This work aimed to develop an anionic cellulose nanofiber (CNF) bio-adsorbent from date palm tree waste and to investigate its removal efficiency compared to cationic methylene blue dye from contaminated water. Date palm pulp was first prepared from date palm leaves through acid hydrolysis using H_2_SO_4_, followed by hydrolysis in a basic medium using KOH, in which the process completely removed the components of hemicellulose, lignin, and silica. To obtain anionic CNF, the resulting pulp was further treated with H_2_SO_4_, followed by centrifugation. Biogel formation of the CNF suspension was promoted by sonication, where its removal efficiency of methylene blue dye was studied as a function of dye concentration, temperature, contact time, and pH value. In this work, we investigated two isotherms, i.e., Langmuir and Freundlich. The Langmuir model’s consistency with the experimental data suggests that the adsorption of methylene blue dye onto CNF is monolayer and surface-limited. The reported maximum removal efficiency of 5 mg/g at 60 °C indicates the optimal temperature for adsorption in this specific case. Additionally, a pseudo-second-order model and Elovich model were also utilized to obtain a better understanding of the adsorption mechanism, in which we found not just physical adsorption but also an indication of a chemical reaction occurring between methylene blue dye and CNF. According to the results, that pseudo-second-order model’s consistency with the experimental data suggests that the adsorption of methylene blue (MB) onto CNF is rate-limiting step involving chemisorption between the two. The study reveals that CNF adsorbents derived from renewable natural waste sources such as date palm leaves can be effective in removing cationic contaminants such as methylene blue dye.

## 1. Introduction

The rapid development of various industrial activities has created pressing environmental problems and prompted an urgent need to develop sustainable technologies to remedy these problems. In the case of water pollution problems, the use of natural sources for water treatments has gained a great deal of interest as these treatments can be sustainable and low cost. The existing pollutants in water from industrial activities are diverse, including organic and inorganic particles, chemicals (e.g., detergents, pesticides, heavy metals, per- and polyfluoroalkyl substances (PFASs), and dyes), biomolecules (e.g., pharmaceuticals), and micro-plastics [1]. In addition to chemical pollution [2], natural pollution from events such as wind dust, emissions of volatile organic compounds from plants, and volcanic eruptions that can greatly alter the properties of water has also been recognized as posing notable environmental challenges [3].

In this study, we aimed to demonstrate a potentially low-cost material to remedy contaminant problems involving azo dyes in the textile, printing, and petroleum industries [1,2,3,4,5]. Azo dyes are commonly used due to their strong binding ability with substrates (e.g., natural and synthetic fibers), such that the resulting colors will not be affected by light, washing, oxygen, acids, or bases [4]. The characteristic functionality of azo dyes [5] is thanks to the presence of the n-n chromophoric group [6,7]. Among the different remediation techniques to remove azo dyes from water, the adsorption approach is considered the simplest and most energy-efficient [8], and cost-efficiency can be greatly improved by the development of low-cost adsorption materials [9]. In the current study, methylene blue was chosen as a model azo dye, which has been found in large amounts in many regions around the globe when safe environmental practices have not been strictly followed.

A promising candidate for a low-cost adsorption material is a nanostructured bio-adsorbent derived from natural biomass sources, such as relatively underutilized agriculture residues, which are abundant, sustainable, and environmentally friendly [10,11,12] Several bio-adsorbents have already been demonstrated for the purification of polluted water resulting from the paper industry [13], leather tanning [14,15], and the food processing, plastics, cosmetics, rubber, printing, and dye manufacturing industries [16,17,18,19,20]. Owing to its high adsorption capacity for a variety of pollutants in aqueous solutions, the potential for the utilization of activated carbon is widely acknowledged among bio-adsorbents [20]. Its high adsorption is mainly due to the presence of heteroatoms like oxygen, nitrogen, and sulfur on the surface, which enables adsorption interactions through various interactions such as hydrogen bonding, π–π interactions, and dipole–dipole interactions [21,22]. There are two fundamental mechanisms by which substances adhere to the surface of an adsorbent, namely, chemisorption and physisorption. Chemisorption involves the formation of strong chemical bonds between the adsorbate and the adsorbent. The chemical bonds formed in chemisorption are often difficult to break, making the process largely irreversible under normal conditions. In contrast, physisorption is characterized by weaker interactions, such as van der Waals forces. Due to its weaker interaction, physical adsorption is often reversible, allowing the adsorbate to be easily desorbed from the adsorbent surface under appropriate conditions.

The adsorption ability of bio-adsorbents derived from agricultural residues can involve both physical and chemical adsorptive interactions (if the adsorbent surface is ionically charged). The main physical interactions involve van der Waals forces, hydrogen bonds, polar interactions, π-π interactions, etc. The conversion of natural biomass feedstocks into effective bio-adsorbents requires proper treatments. For the production of activated carbon, the approach usually involves the use of pyrolysis to treat dense biomass feedstock (e.g., coconut shell and wood), followed by an activation step [23]. However, such an approach is generally not economically beneficial to treat porous biomass feedstock (i.e., leaves, agricultural stalks…). As all plant-based biomass feedstocks contain linear cellulose polymers with β-1,4-linked D-glucose repeating units, the recently developed treatments to produce anionic CNF are appropriate to produce a different kind of bio-adsorbent with abundant hydroxyl (and other functional) groups, which can create intermolecular and intramolecular interactions to remove contaminants [24]. These adsorbents are typically in the suspension state, and thus they can be used more as coagulants/flocculants than solid adsorbents (e.g., activated carbon) for water purification.

The biomass feedstocks most suitable for low-energy anionic CNF production include leaves, grasses, natural fibers, and marine algae [25]. This is because these feedstocks have a relatively loose structure, high porosity, and low lignin content, where a large surface area can be produced in even mild defibrillation conditions, yielding a high adsorption efficiency. These are several interesting examples in the literature of using agricultural biomass for dye removal. For example, the performance of several cellulose-based aerogels prepared from Pomelo fruit to adsorb different dyes (i.e., methylene blue, malachite green, rhodamine 6G, rose bengal, and methyl orange) has been studied. These aerogels include cellulose (CA), graphene oxide (GOA), and a cellulose–graphene oxide composite (CGA). It was found that the adsorption efficiency of CGA aerogels (98%) against methylene blue dyes is higher than those of GOA (95%) and CA (74%) aerogels [26]. 

The adsorption of methylene blue dye in an aqueous solution using (polyester, nonwoven, modified by cross-linking) carboxymethyl cellulose and synthesized ZIF-8 was investigated [27]. The study involved the change in dye concentration and contact time in both batch and continuous fixed-bed conditions. The results showed that there was a slight variation in performance between the continuous and batch systems. For the continuous system, an adsorption capacity of 0.824 mg/g was observed, whereas for the batch system, it was found to be 0.829 mg/g. Both results were obtained at initial concentrations ranging from 2 to 8 mg/L. When increasing the initial dye concentration from 2 to 8 mg/L, the adsorption capacity was increased by about 80 and 90% in the batch and continuous systems, respectively. In another example, regenerated cellulose (RC) was used as an adsorbent for wastewater streams containing varying dyes. The RC adsorbent was produced by the cellulose dissolution waste stream, where the maximum adsorption capacity of RC against crystal violet was 398.41 mg g^−1^ and its maximum adsorption capacity against methylene blue was 367.65 mg g^−1^ [28]. Finally, a hydrogel with an average particle size of around 14.9 μm was synthesized from carboxymethyl cellulose nanocrystals and L-cysteine, where the composite hydrogel was used as an adsorbent for methylene blue removal. The maximum adsorption capacity of this hydrogel system could reach 756 mg/gm at pH = 11 [29].

In this study, we aimed to demonstrate the production of CNF using a typical method from renewable natural resources such as date palm leaves, which are abundant in the local environment but often considered waste. The resulting anionic CNF was tested as a bio-adsorbent for dye removal using MB as a model dye contaminant. The optimal condition for the maximum removal efficiency was evaluated and compared with those for other bio-adsorbents. The broader impact of this study involves waste upcycling and sustainable remediation materials’ development to tackle industrial water pollution challenges.

## 2. Results and Discussion

### 2.1. Characterization of Anionic CNF

**Figure 1:**
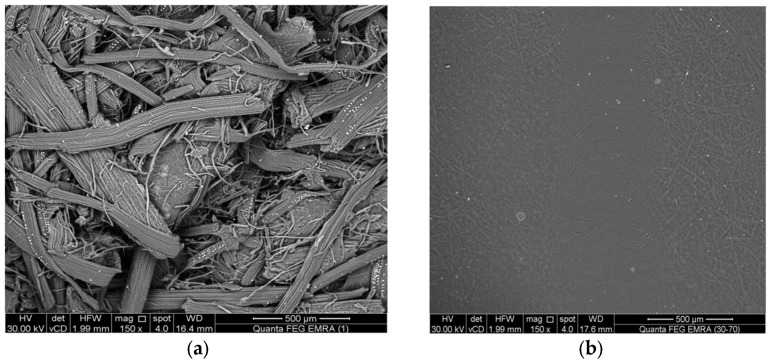
SEM images of (**a**) hydrolyzed cellulose and (**b**) prepared anionic CNF.

The change in the particle structure resulting from the chemical and thermal treatment was studied using scanning electron microscopic images of hydrolyzed cellulose and anionic CNF Figure 1. SEM images were taken at a magnification of 150× to determine the particle distributions and diameters. The SEM images in Figure 1b confirm that the fibers became shorter in length and their width was reduced as a result of the acidic hydrolytic process. It can also be noted that the radius of the fibers was up to 30 microns in hydrolyzed cellulose, and that the size decreased with progress of the hydrolytic process until it reached a size of less than 60 nanometers with a highly homogeneous shape. The presence of the characteristic two peaks around 2θ = 22.6° and 16° indicates the finger print for the cellulose nanostructure [30], and the CNF image shows the appearance of crystallinity in the obtained materials.

**Figure 2:**
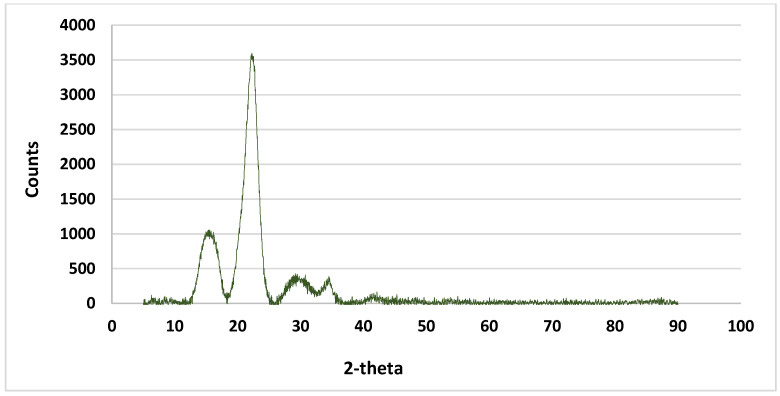
XRD pattern of the nanocellulose fiber.

The change in the ratio between the amorphous and crystalline forms during the chemical and thermal treatment process was studied by X-ray spectrometry. It is well known that natural cellulose is cellulose I. It converts to cellulose II under the effect of an alkaline treatment. Knowing that cellulose ‘I’ is metastable and cellulose ‘II’ is stable, it is possible to form cellulose III and cellulose IV with the progress of the alkaline treatment. The transformation of cellulose I to cellulose II can be investigated by X-ray diffraction. The XRD of the prepared anionic cellulose nanofibers was recorded at the different characteristic 2θ peaks Figure 2, corresponding to the lattice crystal. The two peaks at 2θ = 22.15 and 15.6 are attributed to the amorphous region and cellulosic structure, respectively. It can be seen that the fibers of palm tree leaf are soft and long, and they have a smooth surface Figure 1a.

The degree of crystallinity was calculated according to the following equations:

(1) $$CI(\%)=\frac{A_{crystalinity}}{A_{amorphous}+A_{crystalinity}\;\;}\times 100$$

where ***A****_amorphous_* represents the area under the curve of the amorphous region, and ***A****_crystalline_* is the area under the curve of the sample.

The crystal size was calculated as follows:

L = λk/c β θ(2)

where λ = 0.1540 nm, k represents the correction factor of 0.91, θ represents the diffraction angle in radians, and β represents the full width at half maximum.

**Figure 3:**
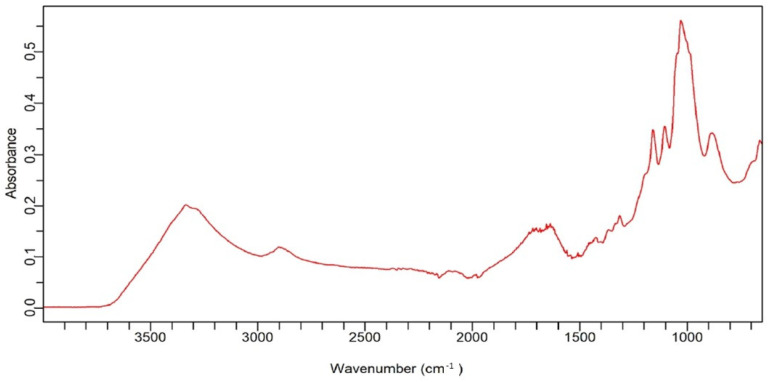
FTIR spectra of anionic CNF.

The FTIR analysis shown in Figure 3 provided valuable information regarding the chemical composition and functional groups present in the prepared CNF. The broad spectral band in the range of 3105–3450 cm^−1^ is indicative of O-H stretching vibrations, which are typically associated with the hydroxyl groups present in cellulose [31]. The peak observed around a wavenumber of 2900 cm^−1^ is attributed to C-H stretching vibrations, which are characteristic of C-H sp^3^ hybridization. The absorption peak around 1646 cm^−1^ is assigned to O-H bending vibrations in the CNF structure, as well as to water molecules absorbed by CNF. At 1100 to 1200 cm^−1^ is an absorption peak due to C–O–C asymmetric stretching vibrations in the glycoside linkages between sugar units in cellulose nanofibers. It confirms the presence of these polysaccharides in the prepared anionic CNF and is a strong indication of a polysaccharide backbone structure [32,33].

**Figure 4:**
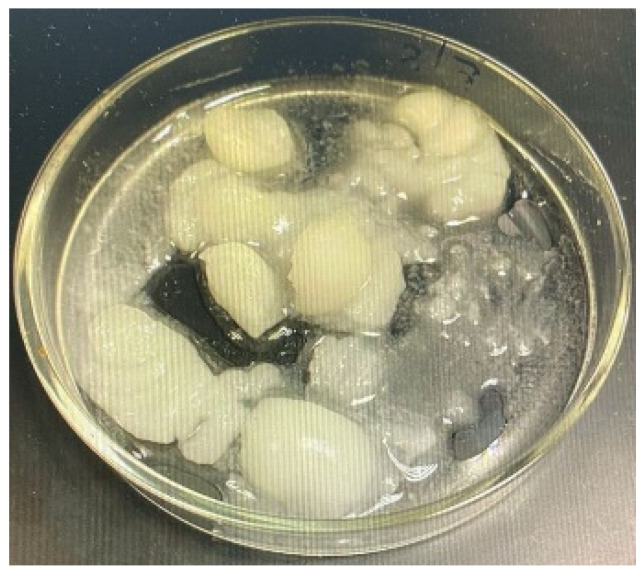
Sample of the obtained nanocellulose.

It is clear from Figure 4 that the sample was a gel-like suspended solution, which confirms that we achieved the characteristic gel generation of a nanomaterial.

### 2.2. Equilibrium Time Experiments

**Figure 5:**
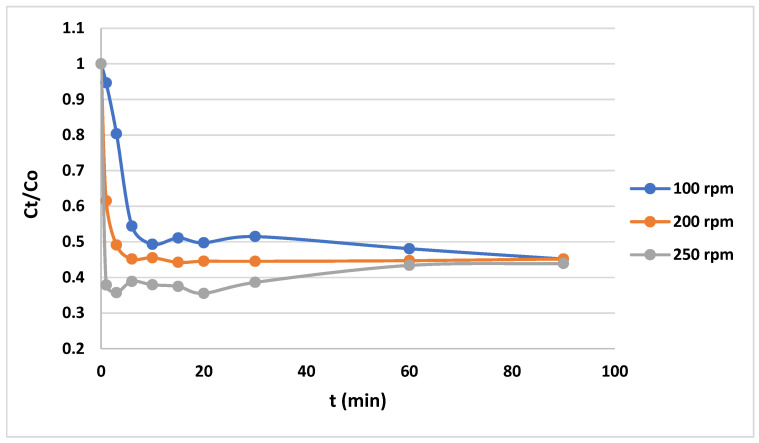
Adsorption rate of MB on anionic CNF at C_o_ = 100 ppm and varying stirrer speeds.

**Figure 6:**
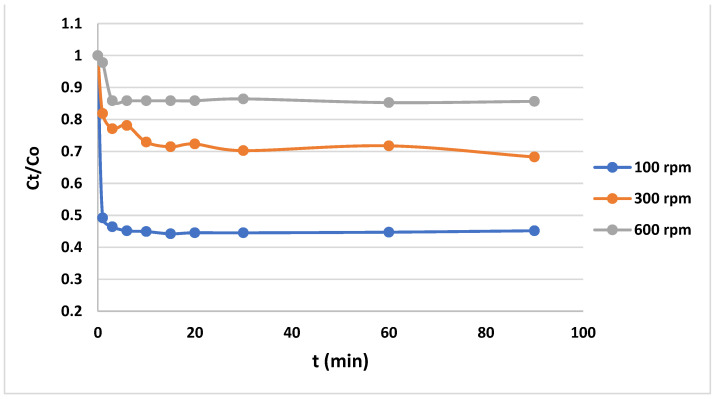
Adsorption rate of MB on anionic CNF (at 200 rpm and varying initial concentrations).

Regarding the equilibrium time, Figure 5 and Figure 6 indicate that it takes 40 min for the dye/anionic CNF to reach equilibrium.

### 2.3. The Effect of pH

**Figure 7:**
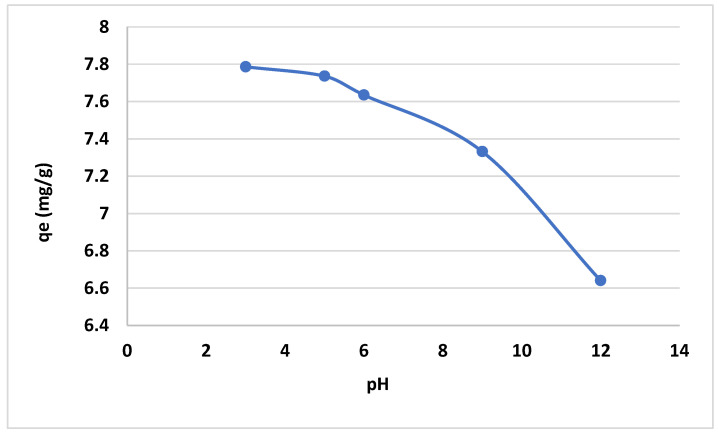
pH vs. q_e_ for adsorption of dye on anionic CNF.

The effect of the pH on the removal efficiency was investigated in the range between 2 and 12. The use of 0.5 M NaOH and 0.5 M HCl is a frequent practice to adjust the pH value. It is apparent from the data shown in Figure 7 that the adsorption efficiency of the anionic CNF for the positively charged dye increased with pH values from 3 to 5. The maximum dye uptake was at a pH of 6. This phenomenon is due to enhanced electrostatic attractions at this pH level.

### 2.4. The Effect of the Dye Equilibrium Constant

Understanding adsorption characteristics is crucial for optimizing the use of the nanomaterial in practical applications. For that purpose, we studied the equilibrium adsorption isotherm, which is a graphical representation of the amount of dye adsorbed (often expressed in mg of dye per g of adsorbent) against the equilibrium concentration of the dye in the solution. We investigated the isotherm at various temperatures (20 °C, 30 °C, and 40 °C). The results showed that the maximum adsorption capacity of the dye on the surface of the anionic CNF was 4.25 mg/g at 20 °C.

### 2.5. The Effect of Temperature

**Figure 8:**
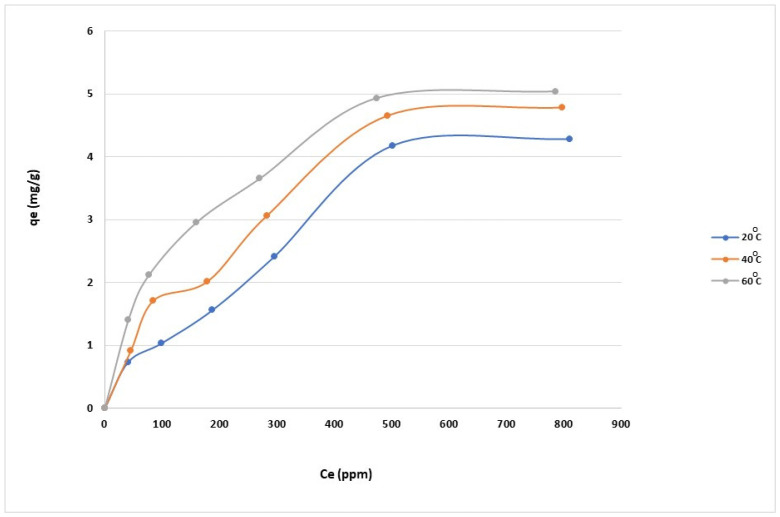
Adsorption of MB on anionic CNF at different temperatures.

The impact of temperature on adsorption was examined at temperatures ranging from 20 °C to 60 °C. The data in Figure 8 show that the adsorption capacities increased at higher temperatures. Since the adsorption process is exothermic, increasing the temperature of the adsorption of dye on CNF is favorable. In this work, the maximum dye removal was recorded at 5 mg/g.

### 2.6. The Adsorption Mechanism

**Figure 9:**
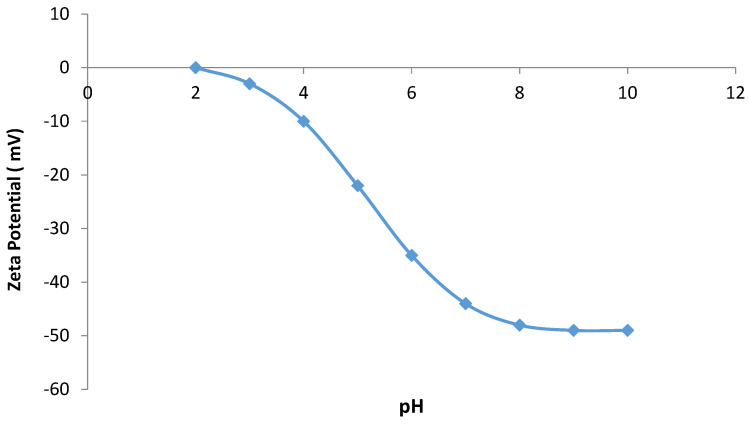
Variation in zeta potential versus pH for adsorption of dye on anionic CNF.

The variation in the zeta potential of the anionic CNF concerning pH was studied to determine the surface charge of the adsorbent. The results of the variable zeta potential as a function of pH are shown in Figure 9. The dye adsorption on anionic CNF is influenced by the pH of the solution. The charge on the surface of the anionic CNF varies with the pH change, which results in variations in removal efficiency. Negative charges on the surface of the anionic CNF increase at higher pH values; therefore, the molecules of the cationic dye will be more attracted to the surface, and accordingly, the dye removal efficiency will be enhanced.

### 2.7. Equilibrium-Adsorption Isotherm

The results data showing the equilibrium-adsorption isotherm are plotted in Figure 2. The isotherm illustrates the relationship between the amount of adsorbed MB on the CNF and the equilibrium MB concentration in the solution under the experimental conditions. That the maximum adsorption capacity of MB on anionic CNF is shown to be 4.25 mg/g at 20 °C. This result suggests a strong interaction between the dye molecules and the surface of the nanofibers at this temperature.

**Figure 10:**
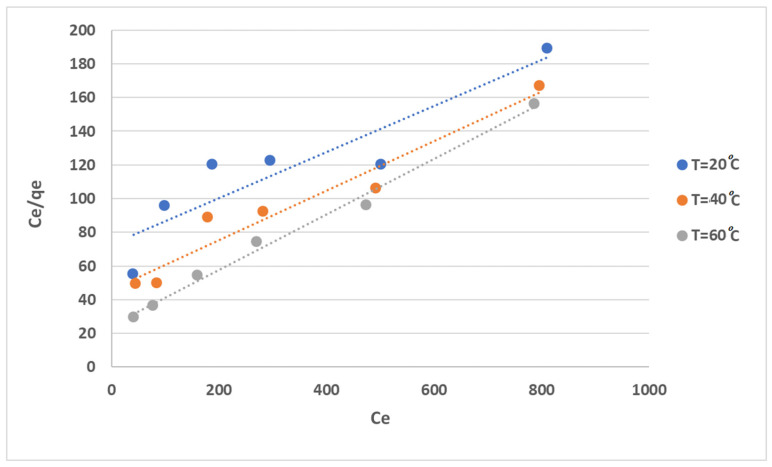
Langmuir isotherm fitting the experimental data of MB adsorption onto anionic CNF.

The use of equilibrium adsorption isotherms, such as the Langmuir and Freundlich models, to analyze the adsorption process is a well-established method. The determination of the Langmuir isotherm constants (K and b) and the Freundlich isotherm constants (KF and n) through linearized plots provides a quantitative measure of the adsorption process. For instance, plotting Ce/qe versus Ce using the linearized Langmuir model, shown in Figure 10, allows for the determination of the equilibrium-constant parameters for the adsorption process based on the calculated slope and intercept. The Langmuir equilibrium parameters, K and b, of the system are listed in Table 1. The results suggest that the Langmuir model satisfactorily explained the experimental data According to the value of the adjusted coefficient of determination R^2^, the Langmuir model sufficiently explained the experimental data.

**Figure 11:**
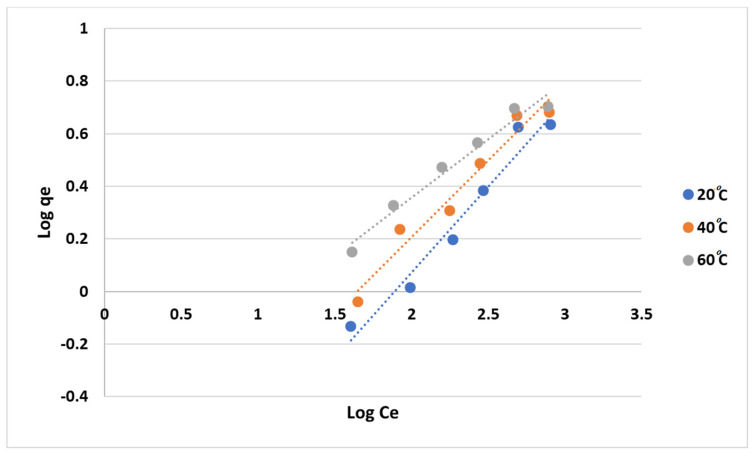
Freundlich isotherm fitting the experimental data of MB adsorption onto anionic CNF.

Similarly, the Freundlich isotherm was also used to study the adsorption mechanism, and the equilibrium-constant parameters, i.e., KF and n, were obtained from the slope and intercept of the linearized Freundlich model. Figure 11 shows that the Freundlich model better fits the experimental data than the Langmuir model. This was also supported by comparing R^2^ values, as shown in Table 1. However, the observation made on both isotherms was intriguing as the Langmuir model fits the experimental data better at higher temperatures, while the Freundlich model provides a better fit at lower temperatures. This could suggest that the adsorption mechanism of MB onto anionic CNF may transition from a monolayer adsorption process (Langmuir) at higher temperatures to a multilayer or heterogeneous adsorption process (Freundlich) at lower temperatures. Another observation was a value of n that was greater than unity, indicating that MB was readily adsorbed on anionic CNF [34]. 

### 2.8. Kinetic Studies

As shown in Figure 5 and Figure 6, the amount of MB in the wastewater gradually decreases due to it binding to the anionic CNF surface. The initial concentration of methylene blue in the wastewater is one of the factors influencing the adsorption of MB onto the anionic CNF from wastewater. In general, lower initial concentrations of MB show faster concentration decreases than higher initial concentrations. This is because at lower initial MB concentrations, less binding sites are occupied by MB molecules; thus, there are more remaining vacant binding sites available for adsorption. This results in a higher rate of adsorption. Because of this higher rate of adsorption, the concentration of MB drops quickly [35]. Agitation plays a crucial role in the kinetics of adsorption by influencing the mass transfer of MB on the anionic CNF. At faster agitation speeds, the increased turbulence within the solution reduces the boundary layer thickness around the adsorbent particles, enhancing the mass transfer rate. Thus, the MB concentration decreases more quickly. On the other hand, mass transfer may end up being the rate-limiting step in the adsorption process at low agitation speeds due to the thicker boundary layer around the adsorbent providing more resistance during the transfer process [36].

#### 2.8.1. Discussion of the Effect of the Chemical Reaction as “A Rate-Controlling Step” Using “Reaction Models” to Describe the Chemical Reaction Mechanism

The use of various kinetic models, such as the “pseudo-first-order”, “pseudo-second-order”, and “Elovich models”, is a robust approach to revealing and explaining an adsorption mechanism that may include a chemical reaction. Here, the results showed that the agitation speed and the initial MB concentration significantly affected the adsorption rate.

**Figure 12:**
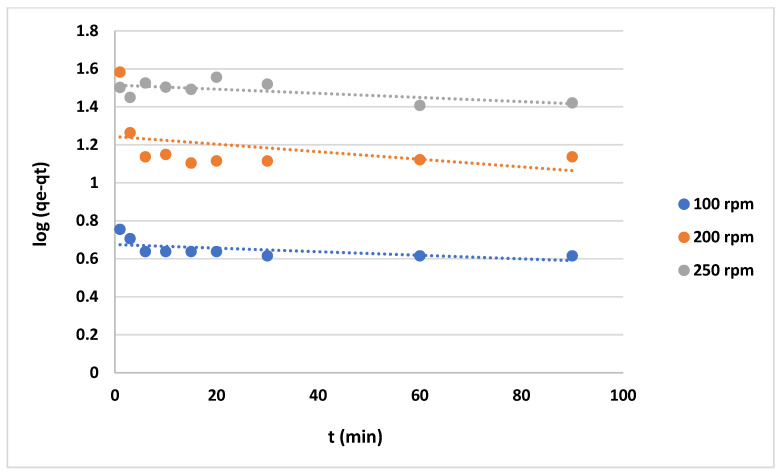
Plots of “pseudo-first-order model” for adsorption of MB on anionic CNF at different agitation speeds.

**Figure 13:**
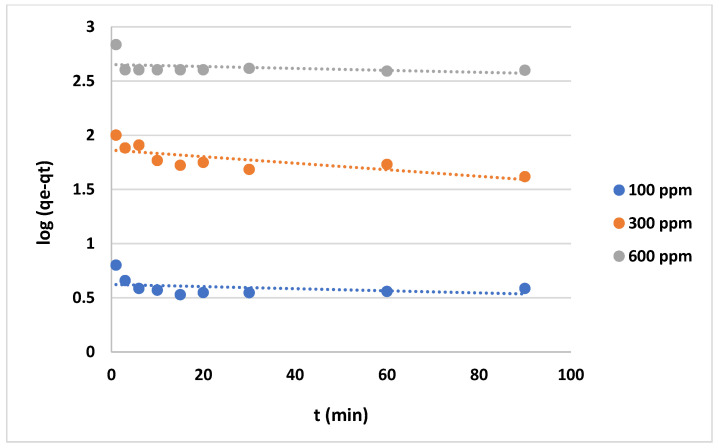
Plots of “pseudo-first-order model” for adsorption of MB on anionic CNF at different initial concentrations.

**Figure 14:**
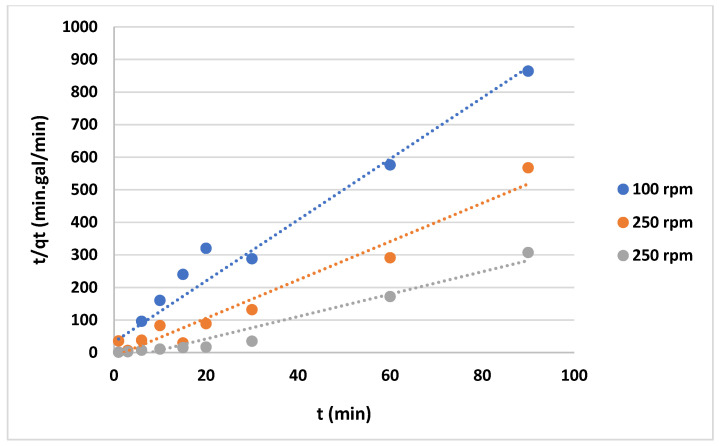
Graphs of the “pseudo-second-order model” for MB adsorption on anionic CNF at various agitation speeds.

**Figure 15:**
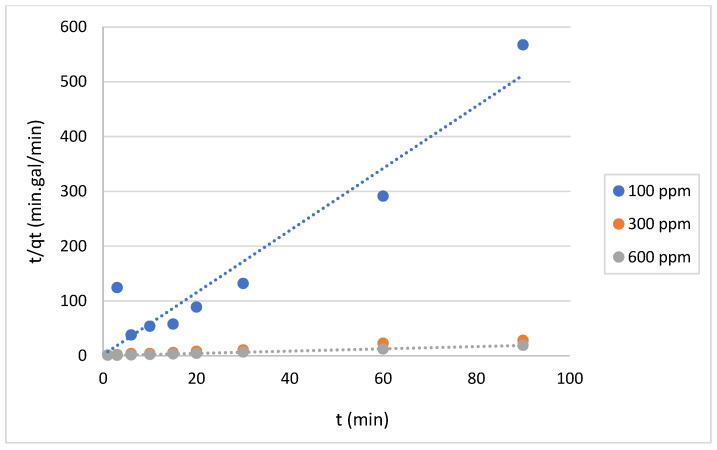
Graphs of the “pseudo-second-order model” for MB adsorption on anionic CNF at different initial concentrations.

**Figure 16:**
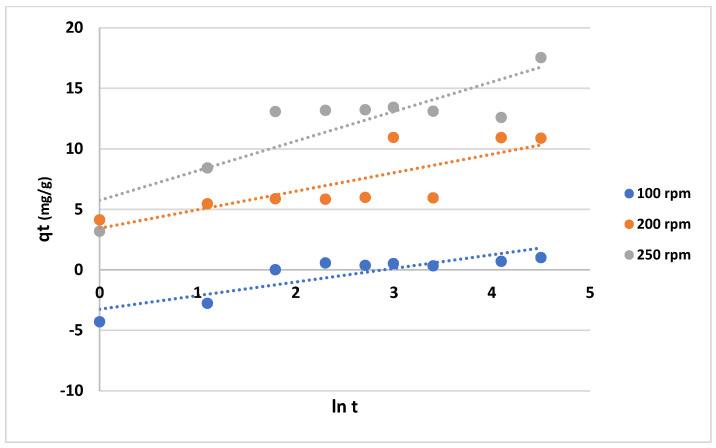
Plots of the “Elovich model” for adsorption of MB on anionic CNF at different agitation speeds.

**Figure 17:**
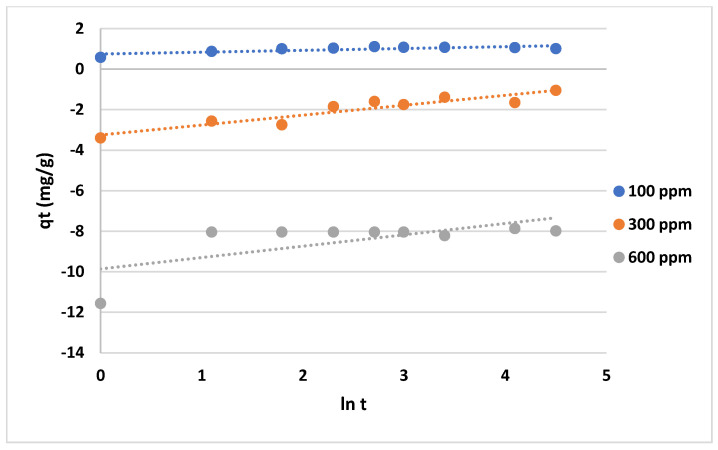
Plots of the “Elovich model” for adsorption of MB on anionic CNF at different initial concentrations.

The data shown in Figure 12 and Figure 13 represent the results for various initial concentrations and agitation speeds for the pseudo-first-order model, while Table 2 lists the corresponding parameters. The correlation coefficient (R^2^-value) suggests very poor fitting between the model and the experimental data. Meanwhile, the data presented in Figure 14 and Figure 15 show the results for the “pseudo second-order model”. The R^2^ value for this model is presented in Table 2, suggesting that the model accurately represents the experimental data. The pseudo-second-order model exhibits even better fitting compared to the Elovich model see Figure 16 and Figure 17. In general, it is clear that both the “pseudo-second-order” model and the Elovich equation have been found to be more suitable for representing the experimental results, as opposed to the “pseudo-first-order model”. Thus, chemisorption could be the mechanism of adsorption that occurs in the adsorption of MB using CNF [37].

#### 2.8.2. Comparison of the Kinetic Models

As mentioned, to ascertain the rate of the chemical reaction between MB and anionic CNF as the prime controlling step, the kinetic adsorption of MB on CNF was examined using a variety of models, including the pseudo-first-order, pseudo-second-order, and Elovich models. A statistical indicator of the linear relationship between two variables is the R^2^. In comparison to the experimental values, the pseudo-first-order model’s correlation coefficient R^2^ was comparatively low, as demonstrated by the results. A low correlation coefficient suggests that there may be other unaccounted factors affecting the adsorption process. In contrast, the second-order equation fit the experimental data well. This suggests that the latter is more suitable for describing the adsorption kinetics of MB on CNF. This is further supported by the higher adsorption-rate constant (k_2_) in the pseudo-second-order model, suggesting chemisorption as the rate-limiting step.

The findings from Gomaa et al. regarding MB adsorption on the CuO@BSS nanocomposite [38] and Fe^3+^ adsorption on a hybrid spongy-like porous carbon material [39] reinforce the utility of the pseudo-second-order kinetic model in describing adsorption processes that are likely controlled by chemisorption. The agreement of the maximum saturation capacity (qe) with experimental data further validates the model’s applicability.

Aside from the above two models, the Elovich equation was also examined against the experimental data. The moderate fit of the Elovich model to the experimental data, indicated by rather higher R^2^ values, suggests that it may also be a relevant model to describe the chemisorption process of MB on anionic CNF. The increase in the Elovich parameter β with agitation speed, while keeping the concentration constant, aligns with the expected decrease in available active sites, corroborating the findings of Gomaa et al. in their study on selective Fe^3+^ adsorption [39].

### 2.9. Adsorption Process Thermodynamics

There are several thermodynamic properties, such as enthalpy (Δ*H*), the standard Gibbs free energy (Δ*G*), and the entropy (Δ*S*) changes, which are essential for understanding the nature of the adsorption process. This can explain the increase in the MB adsorption on anionic CNF with the temperature elevated from 20 °C to 60 °C. The enthalpy change (ΔH) is calculated using the following equation [40]:

(3) $$\ln K=\ln k_{o}+\left(\frac{-\Delta H}{R\;T}\right)$$

**Figure 18:**
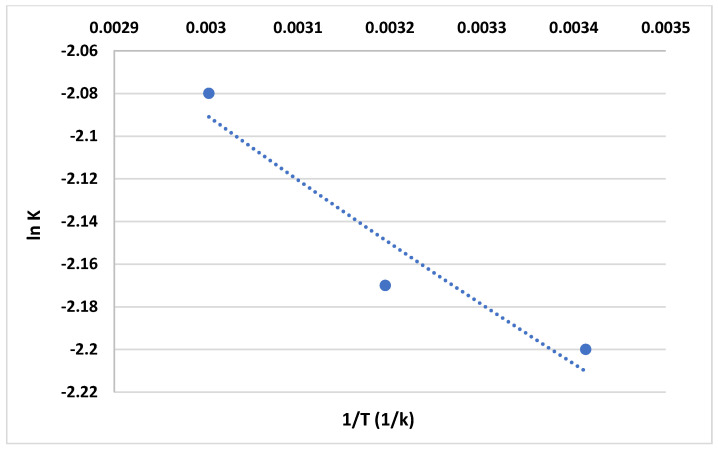
Relationship between ln K and 1/T.

The enthalpy change (Δ*H*) can be determined by constructing a Van’t Hoff plot, which is a graph of the natural logarithm of the equilibrium constant (ln *K*) versus the reciprocal of the absolute temperature (1/*T*), as shown in Figure 18 and also listed in Table 3. Meanwhile, the standard Gibbs free energy change (Δ*G*) is calculated using the Gibbs equation, which relates Δ*G* to the equilibrium constant (*K*), the gas constant (*R*), and the absolute temperature (*T*) [39]:

(4) ΔG=−R T lnK

The equilibrium constant (*K*) can be dimensionless and is calculated using the ratio of the concentration of MB adsorbed by anionic CNF at equilibrium (Cs_m_) to the concentration of MB in the solution at equilibrium (Ce_m_), as expressed in Equation (5) [41]:

*K* = Cs_m_/Ce_m_ (5)

Lastly, the entropy change (Δ*S*) is calculated using the Gibbs–Helmholtz equation, which relates Δ*S* to Δ*H*, Δ*G*, and *T* [41] as follows:

(6) $$\Delta S=\left(\frac{\Delta H-\Delta G}{T}\right)$$

The negative values of ΔH indicate an exothermic adsorption process. Hence, increasing the temperature causes the increase in the MB adsorption on anionic CNF. The sign of Δ*S* can provide information about the disorder or randomness associated with the adsorption process. Negative values of Δ*S*, as shown in Table 3, typically indicate a decrease in disorder upon adsorption. This means the MB-anionic CNF adsorption process may be favorable [42]. The Gibbs equation is a fundamental equation in thermodynamics that provides insight into the spontaneity of a process. A positive Δ*G* indicates a non-spontaneous process under standard conditions.

### 2.10. Comparison of Waste-Palm-Leaf-Derived Anionic CNF as an Adsorbent with Literature-Reported Adsorbents

The utilization of agricultural waste such as palm leaves for the extraction of high-value materials like anionic CNF not only contributes to waste reduction but also offers a cost-effective alternative to traditional raw materials. The fact that the performance of anionic CNF derived from waste palm leaves is on par with other adsorbents mentioned in the literature see Table 4 gives an indication of the potential of this approach. It is encouraging to note that the local and inexpensive nature of the raw material does not detract from the efficacy of the product.

## 3. Conclusions

A highly efficient bio-adsorbent for MB dye was developed by extracting cellulose pulp from the leaves of a date palm tree and transferring it into nanocellulose via multi-chemical treatment. The obtained nanomaterial showed a significant dye removal performance. The temperature and pH are the significant parameters in dye removal. Therefore, the adsorption process is endothermic, and the maximum removal efficiency is at pH = 6 and 60 °C. It was also found that the electrostatic changes in the solution resulting from the pH change greatly affect dye removal. The electrostatic changes at the maximum capacity of the nanocellulose in the adsorption of the dye were 4.25 mg/g at room temperature.

Two equilibrium models, the Langmuir and Freundlich models, were employed. The use of such equilibrium models to describe the adsorption isotherms is a standard approach in adsorption studies. It is noteworthy that the Langmuir model, which assumes monolayer adsorption on a homogeneous surface with a finite number of identical sites, was consistent with the experimental data. This suggests that the adsorption sites on the anionic CNF are uniform and that each site binds to one dye molecule. The highest removal efficiency was obtained at 60 °C and was 5 mg/g.

Kinetic studies involving various models such as pseudo-first-order, pseudo-second-order, and Elovich equations are crucial for understanding the dynamics of the adsorption process. The pseudo-second-order model fitting the experimental data well suggests that the rate-limiting step may be chemical adsorption involving valency forces through sharing or exchange of electrons between the adsorbent and the adsorbate. The Elovich equation further supports the chemical nature of the adsorption, indicating a reaction between the MB dye and the anionic CNF.

Overall, this study’s findings contribute valuable insights on the potential use of renewable natural waste sources for environmental remediation, particularly in the removal of cationic contaminants. They also underscore the importance of understanding the adsorption kinetics and mechanism to optimize the process for practical applications. The use of anionic CNF from date palm leaves as an adsorbent is an innovative approach that combines sustainability with effective wastewater treatment.

## 4. Materials and Methods

### 4.1. Chemicals

Palm tree leaves, as a renewable cellulose-rich material, were collected from a local farm in our region. MB dye was supplied by the Aldrich Chemical Company, Milwaukee, WI, USA. Sodium hydroxide (NaOH); potassium hydroxide (KOH); sulfuric acid (H_2_SO_4_); acetic acid (CH_3_COOH); and sodium chlorite (NaClO_2_) were high-grade laboratory chemicals, purchased from BDH Chemicals. All chemicals were used as received without further purification.

### 4.2. Preparation of Cellulose

A detailed and methodical approach to the preparation of cellulose pulp from date palm leaves was adopted, as explained in this section. This procedure involves several critical steps that ensure the removal of non-cellulosic components such as hemicellulose, lignin, and silica, which are typical in lignocellulosic biomass. Initially, the date palm leaves are thoroughly washed with distilled water to remove dust and impurities, followed with one day of air-drying. They are subsequently cut and ground into smaller pieces, followed by milling to achieve a uniform size of less than 5 mm. The ground fibers are then treated with sulfuric acid under reflux conditions, which helps in hydrolyzing and removing hemicellulose. In this step, 150 g of ground leaf fiber is mixed in a 1 L round-bottomed flask, where 40 g of sulfuric acid is added gradually under reflux for 2 h at 90 °C. The mixture is then filtered and washed using deionized water until a neutral pH is achieved.

After the removal of hemicellulose, an alkaline hydrolysis step is executed, employing 20% KOH solution to break down and remove lignin and silica. This process also involves refluxing, filtration, and thorough washing to reach a neutral pH. A liquid–solid ratio of 10:1 at the same refluxing temperature and time (90 °C for 2 h) is used. The next step is bleaching of the cellulose pulp using a NaClO_2_ solution at an acidic pH adjusted with acetic acid, which helps in removing the remaining lignin and amorphous cellulose. The solution is stirred at 75 °C for 4 h. Finally, the bleached pulp is filtered, washed to neutrality, and dried in an oven at 80 °C until a constant weight is obtained, indicating the removal of all moisture.

### 4.3. Preparation of Anionic CNF

To ensure the production of high-quality CNF, we hydrolyzed never-dried cellulose pulp under controlled acidic conditions, followed by several purification and homogenization steps. Here is the procedure. Initially, the cellulose pulp was hydrolyzed with a 60 wt% H_2_SO_4_ solution at a liquid-to-solid ratio of 1:10. The acid solution was added slowly to the cellulose suspension, and the reaction was conducted at a temperature of 45 °C for 60 min. The hydrolyzed product was extensively washed with cold deionized water to remove any residual sulfuric acid. Next, we centrifuged the mixture at 5000 rpm for 20 min, repeated seven times. The washed suspension was then placed into dialysis tubes with a molecular weight cut-off (MWCO) of 6–8 kDa and dialyzed against deionized water for 48 h to equilibrate to a neutral pH of 6.7–6.9. To ensure a homogeneous dispersion of the CNF suspension, the hydrolyzed product was sonicated at a frequency of 20 kHz for 10 min at a temperature of 10 °C. The ratio of the suspension to water was maintained at 20:1. The sonicated suspension was then centrifuged at 10,000 rpm for 10 min to collect the CNF, followed with drying in an oven at 60 °C for 2 h. The dried CNF was finally stored in sealed containers to prevent contamination and moisture uptake.

### 4.4. CNF Characterization

Scanning electron microscope (SEM) imaging of the prepared cellulose and CNF samples was carried out using a JSM-IT 500 HR (JEOL, Tokyo, Japan) instrument operated at an accelerating voltage of 10 kV. Prior to SEM, the samples were mounted on metal holders and sputter-coated with a thin layer of gold. The coating was applied for 45 min to enhance the electrical conductivity of the sample surface, which is essential for SEM imaging to prevent charging effects and obtain high-quality images.

The X-ray diffraction (XRD) of the different samples was conducted utilizing a D8 Brucker X-ray diffractometer, with Cu-Kα radiation with a wavelength (λ) of 1.54178 Ǻ, operated over a 2θ range of 10–70 degrees with a step size of 0.02 degrees. XRD patterns were collected for the raw material, cellulose pulp, and CNF to investigate their crystalline structures and to identify any changes in crystallinity due to the treatment and processing of the samples.

Corresponding Fourier transfer infrared (FTIR) spectroscopy of different samples (the raw material, cellulose, and CNF) was carried out using a Nicolet 6700 instrument in the transmission mode, where the FTIR spectra were recorded between 4500 and 400 cm^−1^ with a spectral resolution of 4 cm^−1^.

### 4.5. Dye Adsorption Study

**Figure 19:**
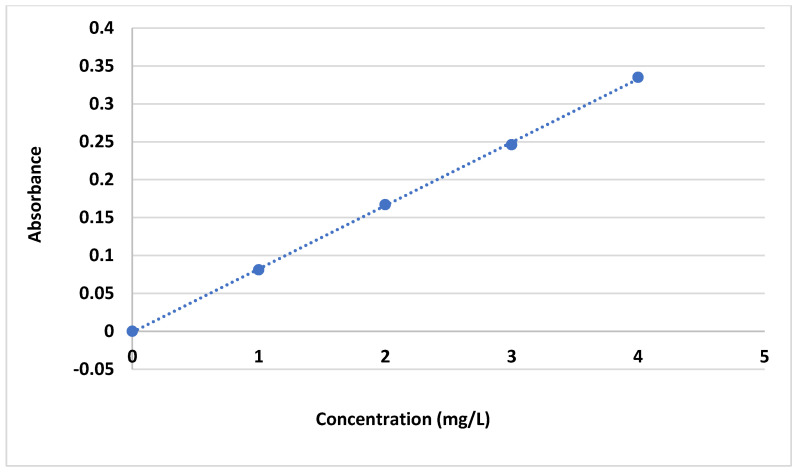
Calibration curve of MB dye.

Before the dye adsorption study using bio-adsorbents, the following light adsorption measurement was first carried out. The spectrophotometer (Spectronic Genesys 5. MPN: 336008, Emeryville, CA, USA) was used to determine the maximum wavelength at which the highest dye absorption efficiency occurred (the max. wavelength was 664 nm for MB). To determine the sensitivity range of the spectrophotometer in measuring the absorbance of MB dye, a calibration curve was first established. The absorbance of MB dye concentrations was measured in the range of 1–4 ppm at the maximum wavelength of 664 nm. Following Beer’s law, a linear relationship between the absorbance and the dye concentration was obtained Figure 19, which allowed for the determination of the MB dye concentration between 1 and 7 mg/L. For higher concentrations, the tested solutions were diluted accordingly.

### 4.6. Experimental Adsorption Equilibrium Isotherm

The adsorption experiments to evaluate the dye removal efficiency of CNF adsorbents were carried out in a 250 mL stoppered conical flask containing 50 mL of dye solution at varying concentrations. The adsorption equilibrium isotherm data were obtained from the batch adsorption experiments at an agitation speed of 250 rpm. The initial dye concentration was prepared by diluting a stock solution with a known concentration of 1000 mg/L. To calculate the amount of dye adsorbed at equilibrium per unit mass of the adsorbent, the following equation was used

(7) $$q_{e}=V\frac{\left(C_{o}-C_{e}\right)}{m}$$

where V (L) is the volume of solution, m (g) is the mass of the adsorbent used, and C_o_ and C_e_ (mg L^−1^) are the initial and final concentrations of dye in the aqueous solution, respectively.

We considered several experimental factors such as the pH of the solution, the initial concentration of the dye, and the contact time. pH influences both the surface charge of the adsorbent and the ionization state of the dye molecules; thus, it can significantly affect the adsorption process. By varying the initial concentration of the dye, the adsorption capacity of the CNF at different concentrations can be studied. Meanwhile, the duration of the interaction between the dye solution and the CNF can determine how quickly the equilibrium is reached and the efficiency of adsorption over time. Figure 8 presents the equilibrium between the amount of adsorbed MB on the CNF and the concentration of MB in the solution.

### 4.7. Kinetic Experiments

It is important to measure the adsorption rate precisely during kinetics experiments. The below procedure was methodically structured to evaluate the influence of the agitation speed and initial MB concentration on the adsorption kinetics. To initiate the adsorption process, 1 g of anionic CNF is added to 50 mL of the MB solution. Initial concentrations of MB are prepared and placed in special bottles. These bottles are then situated in a thermostat-controlled shaker to ensure a constant temperature throughout the experiment, which is crucial for maintaining consistent experimental conditions. The adsorption process is monitored over a period of 90 min. During this time, the agitation speed is controlled and maintained at a specific rate. Typically, the adsorption rate is higher at the beginning due to the abundance of available adsorption sites, and decreases gradually as these sites are occupied, until equilibrium is reached where no further adsorption occurs. At predetermined time intervals during the 90 min shaking period, samples are taken from the shaker. These samples are immediately filtered to separate the CNF from the MB solution, to measure the remaining amount of MB.

The agitation speed plays an important role in the adsorption process. Agitation is needed to mix the solution, which, in turn, affects the mass transfer of the adsorbate to the adsorbent. In our work, the procedure that was previously explained was carried out for various agitation speeds and initial MB concentrations. The adsorption experiments were conducted with agitation speeds set at 100 rpm, 200 rpm, and 250 rpm. These speeds were chosen to represent a range of mixing intensities that could influence the mass transfer rate of MB to the anionic CNF. The resulting data of concentration against time were plotted, as shown in Figure 5. Meanwhile, to assess the effect of the initial MB concentration on the adsorption process, concentrations were varied from 100 ppm to 600 ppm. During these experiments, the agitation speed was consistently maintained at 200 rpm, ensuring that the only variable affecting the adsorption rate was the initial concentration of MB. Similarly, the resulting data of concentration against time were also plotted, as shown in Figure 6.

### 4.8. Adsorption Equilibrium Isotherm Models

The Langmuir and Freundlich isotherms are two commonly employed models for characterizing the adsorption process of contaminants such as dyes on various adsorbents.

#### 4.8.1. Langmuir Isotherm Model

The Langmuir isotherm model is based on the assumption that adsorption occurs at specific homogeneous sites within the adsorbent, and that once a dye molecule occupies a site, no further adsorption can take place at that site. The relation is expressed as follows:

(8) $$q_{e}=\frac{KC_{e}}{1+bC_{e}}$$

The model can be linearized as follows:

(9) $$\frac{C_{e}}{q_{e}}=\frac{1}{K}+\left(\frac{b}{K}\right)C_{e}$$

Hence, a plot of C_e_/q_e_ versus C_e_ provides the equilibrium-constant K and b for the adsorption process, obtained from the slope and intercept of the linear plot.

#### 4.8.2. Freundlich Isotherm Model

The Freundlich isotherm model, on the other hand, assumes a heterogeneous surface with a non-uniform distribution of heat of adsorption over the surface. The Freundlich isotherm is an empirical equation that can be represented as follows:

(10) $$q_{e}=K_{F}C_{e}^{1/n}$$

The linearization of the Freundlich model is expressed as follows:

(11) $$\log\;q_{e}=\log\;K_{F}+\left(\frac{1}{n}\right)\log\;C_{e}$$

The constants, K_F_ and n, are obtained from the intercept and slope of linear plot of log q_e_ against log C_e_.

To validate the experimental results, one would typically fit the experimental data to both the Langmuir and Freundlich isotherm models and determine the corresponding parameters for each model. The goodness of fit is often assessed using statistical metrics such as the coefficient of determination (R^2^) or the chi-square test. The model that best describes the adsorption process is determined by the higher correlation coefficients and the conformity of the model assumptions with the experimental observations.

### 4.9. Kinetic Models

Kinetic models including those in which a chemical reaction is considered as the rate-limiting step were also used to explain the experimental data.

#### 4.9.1. Describing the Batch Adsorption Process Using Reaction Models

Many researchers have used pseudo-first order, pseudo-second order, and Elovich models to study dye adsorption that involves chemical reactions as the rate-limiting step [45,46,47,48,49,50,51,52,53].

##### Pseudo-First-Order Model

In the case of a pseudo-first order model, the adsorption rate of MB on CNF is assumed to be proportional to the amount of adsorbed MB. The model is expressed as follows:

(12) $$\left(\frac{{\mathrm{dq}}_{t}}{\mathrm{dt}})=k_{1}\;(q_{e}-q_{t}\right)$$

where q_e_ and q_t_ are the adsorption capacities (mg/g) of MB at equilibrium and time t, respectively, while k_1_ is the pseudo-first-order adsorption-rate constant (1/min). By integrating Equation (12) and using initial condition of q_t_ = 0 at t = 0, one can obtain

(13) $$ln\;(q_{e}-q_{t})=ln\;q_{e}-k_{1}t$$

##### Pseudo-Second-Order Model

MB adsorption can also be described using a modified second-order pseudo equation. It can be written as

(14) $$\frac{{dq}_{t}}{dt}=k_{2}{\left(q_{e}-q_{t}\right)}^{2}$$

where q_e_ and q_t_ are the adsorption capacities (mg/g) of MB at the equilibrium and time t, respectively. Meanwhile, k_2_ (g/mg·min) is the pseudo-second-order adsorption-rate constant. Equation (14) can be integrated, using the boundary condition of q_t_ = 0 at t = 0 and q_t_ = q_t_ at t = t, and one can obtain the following equation:

(15) $$\left(\frac{t}{q_{t}}\right)=\left(\frac{1}{k_{2}q_{e}^{2}}\right)+(\frac{1}{q_{e}})t$$

As shown, if the data fit the model, one can obtain a linear relationship with a slope of 1/q_e_ and an intercept of ($1/k_{2}q_{e}^{2}$) by plotting (t/q_t_) versus t.

##### Elovich Model

The Elovich equation is expressed as follows:

(16) $$\left(\frac{{\mathrm{dq}}_{t}}{\mathrm{dt}})=œ\;\exp\;(-ß\;q_{t}\right)$$

where q_t_ is the methylene blue amount adsorbed on anionic CNF at time t, œ is the initial MB adsorption rate (mg/g·min), and ß is the desorption constant (g/mg) for a given experimental period. For the simplification of the Elovich equation, assuming ß >> 1, the boundary condition q_t_ = 0 is applied when t = 0, and q_t_ = q_t_ is applied when t = t. Then, Equation (16) becomes

q_t_ = ß ln (œ ß) + ß ln t (17)

To determine the Elovich equations that fit the kinetics, Equation (17) was used to represent the adsorption rate of methylene blue on anionic CNF. Thereafter, constant values can be obtained from the linear slope and intercept of the plot of q_t_ versus ln t.

## Figures and Tables

**Table 1:** Isotherm constants for MB adsorption on anionic CNF.

	Langmuir Isotherm			Freundlich Isotherm		
Temperature (°C)	K (L/g)	b (L/mg)	R^2^	k_F_ (L/g)	n (−)	R^2^
20	0.014	0.002	0.825	0.058	1.529	0.943
40	0.022	0.003	0.94	0.109	1.716	0.959
60	0.041	0.007	0.992	0.295	2.254	0.972

**Table 2:** Kinetic model parameters for MB adsorption on anionic CNF.

Adsorbent Parameters	Pseudo-First-Order		Pseudo-Second-Order		Elovich		
	k_1_ (min^−1^)	R^2^	k_2_ (g/mg min)	R^2^	œ (mg/g min)	ß (g/mg)	R^2^
Initial conc. (mg/L)							
100	−0.001	0.119	17.63	0.921	21.56	0.089	0.597
300	−0.003	0.558	0.0748	0.894	0.049	0.4891	0.874
600	−0.0009	0.12	0.232	0.998	5.3 × 10^−5^	0.560	0.463
Agitation speed (rpm)							
100	−0.0009	0.347	2.697	0.956	0.112	1.124	0.765
200	−0.002	0.151	2.749	0.955	4.417	1.529	0.634
250	−0.0011	0.425	2.695	0.955	11.14	2.445	0.760

**Table 3:** Thermodynamic parameters for the MB adsorption of anionic CNF at different temperature levels.

Temperature (°C)	ΔH (KJ/mol)	ΔG (KJ/mol)	ΔS (KJ/mol K)
20		5.47	−0.0416
40	−6.71	5.56	−0.0392
60		5.69	−0.0373

**Table 4:** Comparison of the prepared anionic CNF with other reported literature results.

Ref.	Adsorbent	Solution	T (°C)	Capacity
	Type			mg/g
[43]	CNF Membrane	Crystal Violet (CV)	25	4
[44]	CNF	Congo Red (CR)	25	6.6
This work	Anionic CNF	MB	25	4.25

## Data Availability

Data are contained within the article.
